# Analysis of influencing factors and equity in hospitalization expense reimbursement for mobile populations based on random forest model: a cross-sectional study from China

**DOI:** 10.3389/fpubh.2025.1626310

**Published:** 2025-08-18

**Authors:** Lisheng Shen, Xinan Lu, Yanyun Zhang, Lin Fei, Bo Dong

**Affiliations:** ^1^Department of Medical Insurance Affiliated Cixi Hospital, Wenzhou Medical University, Ningbo, China; ^2^Department of Respiratory and Critical Care Medicine, Affiliated Cixi Hospital, Wenzhou Medical University, Ningbo, China; ^3^Department of Record Room, Affiliated Cixi Hospital, Wenzhou Medical University, Ningbo, China; ^4^School of Public Health, Zhejiang Chinese Medical University, Hangzhou, China

**Keywords:** migrant population, hospitalization expenses, random forest model, key factors, equity

## Abstract

**Background:**

Continuously improving the accessibility of hospitalization expense reimbursement and reducing the medical expense burden on the migrant population are crucial objectives of China's health insurance system reform. Existing research lacks comprehensive analyses of the current status of hospitalization expense reimbursement for the migrant population, and insufficiently addresses the factors influencing reimbursement and equity. The study aims to identify the key factors influencing the hospitalization expense reimbursement for China's migrant population and to further analyze the equity of this reimbursement.

**Methods:**

Data were obtained from the 2018 China Migrants Dynamic Survey. After data cleaning, a sample of 3,186 individuals who incurred hospitalization expenses was selected for analysis. First, the current status of hospitalization expense reimbursement (occurrence, location, method, and amount) was analyzed using percentages and chi-square tests. Secondly, the random forest algorithm was applied to evaluate the importance of the factors influencing hospitalization expense reimbursement. Third, the regression analysis was used to quantify the key factors. Finally, the concentration index was utilized to assess the equity of hospitalization expense reimbursement for the migrant population and the contribution of key factors to this equity.

**Results:**

Regarding reimbursement rates, 69.83% of the migrant population chose to reimburse hospitalization expenses, while 30.17% still did not. In terms of reimbursement location, 55.69% reimbursed hospitalization expenses at their place of household registration, and 44.31% at their place of inflow. Regarding reimbursement method, 88.36% chose the Basic Medical Insurance System for Urban and Rural Residents, while 11.64% used the Basic Medical Insurance for Urban Employees. The mean of total hospitalization expenses for the migrant population was 3,058.7 (USD), with health insurance reimbursing 1,213.4 (USD) and individuals paying 1,845.3 (USD) out-of-pocket. The health insurance reimbursement ratio was 39.67%, and the out-of-pocket share was 60.33%. The results of random forest analysis identified the key factors affecting whether the reimbursement occurred as: education, health, age, income, and local insurance enrollment. Key factors affecting the level of reimbursement were: health status, insurance type, total medical expenditure, illness status, and mobility scope. Equity analysis revealed pro-rich inequity (favoring high-income groups) in both the probability and level of hospitalization expense reimbursement. Factors contributing to hospitalization cost reimbursement probability inequity, listed in descending order of impact are education (42.3%), income (34.1%), health (12.4%), age (8.2%), and enrollment location (3.0%); factors contributing to the level of hospitalization reimbursement inequity, listed in descending order of impact are health (58.12%), mobility range (21.74%), total healthcare expenditures (9.35 %), type of healthcare coverage (9.28%), and illness (1.51%).

**Conclusion:**

There is still much room for improvement in the reimbursement rate of hospitalization expenses for the migrant population. Future efforts to strengthen protection should: (1) further improve the coordination level of medical insurance to narrow the treatment differences between different regions; (2) encourage migrant populations to enroll locally (in the inflow area) and participate in the Basic Medical Insurance for Urban Employees to increase reimbursement levels; and (3) simplify reimbursement policies, optimize information dissemination channels, and enhance the policy comprehensibility and acceptance to narrow accessibility gaps.

## Introduction

The term “migrant population” refers to individuals residing outside their household registration (hukou) location (1, 2). China has one of the largest migrant populations around the world. According to the National Bureau of Statistics of China, the number reached nearly 380 million in 2020, an increase of 150 million (nearly 70%) since 2010 (3). It is undeniable that the migrant population has injected impetus into local development and has gained significant development opportunities for themselves; however, they are still unable to enjoy the same social support and security as local residents, facing marginalization (4, 5). Studies have indicated that most of the migrants have lower education levels, work in labor-intensive jobs under poor conditions (6), which puts them at higher risk of occupational diseases, infectious diseases, sexual, and maternal health problems, as well as psychological problems (7, 8). Consequently, they are more likely than the locals to forgo needed health services, resulting in higher health losses and worse health outcomes (9–11).

On the other hand, China's unique medical insurance system contributes to unequal health effects across insurance types for migrants. The system is multi-layered, with the basic medical insurance as the core, supplemented by medical assistance and commercial medical insurance (12, 13), of which the basic medical insurance comprises Basic Medical Insurance System for Urban and Rural Residents (BMISURR) and Basic Medical Insurance for Urban Employees (BMIUE). These different types of medical insurance systems cater to different participants, who differ not only in occupational overage but also in benefits. According to the 2018 Statistical Snapshot of the Development of the Medical Security Career released by China's National Bureau of Medical Security, the average hospitalization expense for BMIUE participants was 2,731 (USD), with an 81.6% policy-covered reimbursement rate; for BMISURR participants, it was 1,606.46 (USD), with a reimbursement rate of 65.6% for hospitalization expenses within the scope of the policy (14). Some studies have confirmed that participation in the BMIUE will yield higher reimbursement rates and more healthcare access for migrants compared to the BMISURR (15–17). Furthermore, enrollment location creates unequal protection the migrant population. China's hukou-based, voluntary participation system (18) means enrollment location often differs from residence. Financial constraints also lead some migrants (estimated at ~10%) to forego insurance altogether (19). Low coordination levels and fragmented territorial management reduce insurance risk-sharing capacity and complicate reimbursement procedures for migrants seeking care outside their hukou location. Migrants often incur extra time and expenses, needing to pay upfront for cross-province care, making reimbursement more difficult compared to locals (20), facing more complex procedures and higher expenses (9).

To alleviate the medical burden on the migrant population and promote the improvement of their health, China has strengthened its medical protection for the migrant population, including the implementation of direct settlement for cross-province care, and the establishment of a nationally unified platform for the provision of medical insurance services, which have had a positive impact on the reimbursement and treatment protection. However, low overall insurance coordination levels and policy differences across regions and insurance types persist. Migrants are still confronted with challenges transferring insurance relationships, low reimbursement rates, and often need to prepay for out-of-area care (21, 22). Therefore, reducing reimbursement barriers and promoting equitable access to medical insurance for migrants remains a critical issue.

Research on hospitalization expense reimbursement for the migrant population in China falls into two categories: the impact of medical insurance on hospitalization expenses, and factors influencing reimbursement. With regard to the former, studies have shown that medical insurance helps reduce expense burden of the migrant population (15), with differences between types: BMIUE offers higher reimbursement than BMISURR (16, 17). In addition, the enrollment location also matters; reimbursement levels are significantly lower when enrolled at the household registration (hukou) location compared to the inflow location (23). With regard to the latter, the research has shown that the factors such as personal characteristics (gender, age, and marital status), socio-economic characteristics (income, education level, and household registration status), health insurance characteristics (location and type), and health status are key determinants of expense burden and reimbursement (24–26). Regarding equity in healthcare utilization, current studies mainly focus on analyzing the equity of the utilization of outpatient services and inpatient services for the migrant population (16, 27), and there is a lack of studies on the equity of the reimbursement of inpatient expenses from the perspective of expenses.

From the above studies, it can be seen that relevant scholars have paid extensive attention to the hospitalization expenses of the migrant population, however, there is still room for further expansion of the relevant studies. First, the research content is relatively narrow, focusing mainly on reimbursement status and influencing factors, with less attention to equity. Second, methodological expansion is required. Most of the existing studies use logistic regression to analyze the degree of impact of different factors on hospitalization expenses, but lack methods to measure relative importance of different factors. Based on this, this study takes the hospitalization expenses of the migrant population as the research object and uses representative national survey data (China Migrants Dynamic Survey) to explore the key factors influencing hospitalization expense reimbursement for the migrant population in China and their equity, aiming to provide a basis for further improving the migrant population's expense protection policy and reducing their burden.

Compared with existing studies, this research contributes in several ways: first, the research uses data from a large-scale micro-survey dedicated to the migrant population in China to comprehensively analyze the current situation of hospitalization expense reimbursement from multiple dimensions, enhancing the understanding of the expense burden at a holistic level. Second, while current studies typically explore independent or cross-influences of factors, they lack comparison of the degree of importance of different variables. This study used the random forest model to measure the importance of different factors, and further screened out the most critical factors, enabling more accurate evaluation. Third, this study analyzes equity in hospitalization expense reimbursement and quantifies the contribution of key factors to inequity. This not only enriches equity analyses but also provides data and theoretical support for developing targeted interventions to equalize the expense burden.

## Research methodology

### Theoretical framework

**Figure 1:**
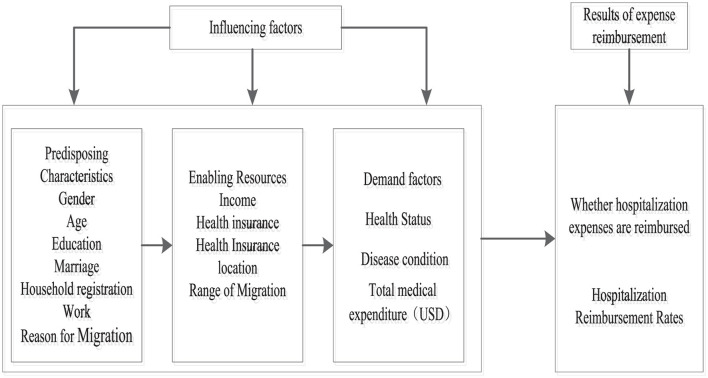
Framework for analyzing the behavior of hospitalization expense reimbursement for mobile population based on Andersen's model.

The Andersen Behavioral Model provides a robust theoretical framework for understanding determinants of hospitalization expenses and their inequities among the migrant population. Widely used in healthcare service research (28, 29), it has also been applied to analyze migrant populations' utilization of public health services (30, 31). The model reveals the relationship between situational characteristics, individual characteristics, health behaviors, and health outcomes, integrating multiple factors within a multidimensional framework to analyze health behaviors. It effectively explains the multidimensional factors affecting the utilization of health services by different groups. These influencing factors can be classified into three categories: (1) Predisposing characteristics, which indicate the tendency to use health service, are the socio-cultural tendency characteristics of individuals prior to becoming ill or seeking health service utilization, and are not directly related to health service utilization, including age, gender, occupation, and education. (2) Enabling resources, which are factors that indirectly affect health service utilization, including income, health insurance, and accessibility of health resources, refers to an individual's ability to access health services and the availability of health resources in the community and household. (3) Need factors, which reflect an individual's subjective perception of health services and actual health status, encompass both perceived need (subjective judgment of disease and health) and assessed need (objective professional assessment). Adapting this model and considering relevant literature (32–34), this study constructs an analytical framework for influencing factors of hospitalization service reimbursement for China's migrant population across propensity, enabling, and need dimensions, as shown in Figure 1.

### Data sources

This study utilizes data from the 2018 China Migrants Dynamic Survey (CMDS), a nationally representative cross-sectional survey of the domestic migrant population conducted annually since 2009 by China's National Health Commission (35). The CMDS is recognized for good representativeness and low sampling error (36). It employs a stratified, multistage, scale-oriented probability proportional of size (PPS) method as the sampling method to select the sample. Rigorous methods ensured data quality, including scientific questionnaire design, enumerator training, survey supervisors verifying questionnaires, and quality checks via telephone callbacks.

**Figure 2:**
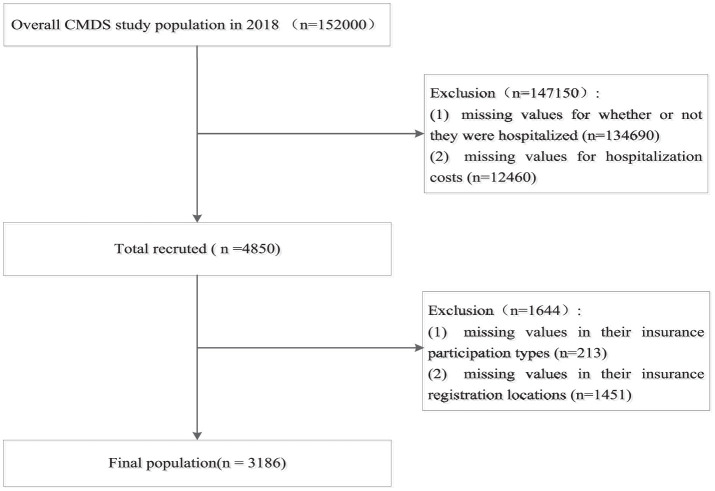
Flow chart of sample inclusion and exclusion.

The CMDS collected information from 152,000 migrants aged 15 or older, residing in their current location for over 1 month without local household registration, across 32 provincial-level administrative regions. The survey covered comprehensive information on the demographic and socioeconomic characteristics, health status, health service utilization, and healthcare expense for the respondents and their household members; for more details about the sample, see other literature (37). After processing missing values, the final analytical sample size was 3,186. The sample inclusion and exclusion process is shown in Figure 2.

### Variables selection

#### Dependent variables

In this study, the dependent variable is hospitalization expense reimbursement of the migrant population, comprising two dimensions: (1) whether reimbursed: measured by “Was your hospitalization expense reimbursed?” (Yes = 1, No = 0). (2) Reimbursement level: measured by the reimbursement rate (reimbursement amount ÷ total hospitalization expenses). Here, the reimbursement amount is calculated as the total hospitalization expenses minus the individual's out-of-pocket payment. The total hospitalization expenses are measured using the question: “How much did you spend in total on this hospitalization?”

#### Independent variables

Based on the theoretical framework and data availability, control variables were selected to adjust for confounding effects, categorized as:

Predisposing characteristics: Gender (Female = 0, Male = 1), Age (calculated from interview year minus birth year), Education (ordinal: Illiteracy = 1, Primary school = 2, Junior high = 3, Senior high = 4, University and above = 5), Marital status (Unmarried/Divorced/Widowed = 0, Married/Remarried/Cohabiting = 1), Occupation (No occupation = 0, Employed = 1), Household registration status (Rural = 1, Urban = 2), Reason for mobility (Family = 1, Work = 2, Other = 3).

Enabling resources: Income level (provincial household income percentile: ≤ 20th, 20–39th, 40–59th, 60–79th, ≥80th), Health insurance enrollment location (Not enrolled locally = 0, Enrolled locally = 1), Health insurance type (BMISURR = 1, BMIUE = 2), Mobility scope (Inter-county = 1, Inter-city = 2, Inter-provincial = 3). Mobility scope is categorized under enabling resources as it primarily affects health service accessibility and availability.

Need factors: Health status (Unhealthy =1, Basically healthy = 2, Healthy = 3), Illness (“In the past year, did you have any disease or physical discomfort?” No = 0, Yes = 1). Total hospitalization expenditure ( ≤ USD 1,221 = 1, USD 1,221–2,442 = 2, ≥USD ≥2,442 = 3) was also included as a control covariate due to its influence on reimbursement choices and levels. Variable definitions and assignments are shown in Table 1.

**Table 1:** Variable selection and definitions.

**Variables**			**Definitions**
Dependent variables		Whether hospitalization expenses are reimbursed	No = 1; Yes = 1
		Reimbursement level for hospitalization expenses	Health insurance reimbursement as a percentage of total hospitalization costs
Independent variables	Predisposing characteristics	Gender	Female = 0
			Male = 1
		Age	15–30 = 1
			31–45 = 2
			46–60 = 3
			61+ = 4
		Education	Illiterate = 1
			Primary school = 2
			Junior middle school = 3
			Senior middle school = 4
			University/college = 5
		Marriage status	Unmarried = 0
			Married = 1
		Employment	Unemployed = 0
			Employed = 1
		Household registration	Rural household registration = 0
			Urban Account = 1
		Reasons for migration	Family = 1
			Work = 2
			Others = 3
	Enabling resources	Range of migration	Intercounty = 1
			Intercity = 2
			Interprovince = 3
		Household income ranking	Lowest (< percentile 20) = 1
			Lower (percentile 20–39) = 2
			Middle (percentile 40–59) = 3
			Higher (percentile 60–79) = 4
			Highest (≥percentile 80) = 5
		Type of health insurance	BMISURR = 1
			BMIUE = 2
		Health Insurance location	Household registration = 0
			Place of inflow = 1
	Demand factors	Health status	Unhealthy = 1
			Basically Healthy = 2
			Healthy = 3
		Total medical expenditure (USD)	≤ 1,221 = 1
			1,221–2,442 = 2
			≥2,442 = 3
		Disease condition	No = 1; Yes = 1

In order to improve international comparability and ease of understanding, in the study, we converted the cost of RMB to 2018 PPP-adjusted US dollars using the PPP conversion factor of 4.0941 CNY per international dollar from the World Bank.

### Statistical methods

#### Random forest model

The random forest model offers advantages including comparing variable importance, high accuracy, efficiency, stability, and reliable results (38). Compared with more complex models like XGBoost, the random forest model is easier to understand and implement, reduces the overfitting risk, and captures complex interactions between features (39, 40), and it has been widely used in medicine, public health, and other fields (41, 42). Some studies use this model to analyze issues related to health service utilization of the Chinese migrant population (43). Drawing on similar studies, this study used the random forest model to measure the importance of factors influencing hospitalization expense reimbursement for China's migrant population.

The random forest algorithm consists of multiple decision trees using bagging algorithm. It randomly samples data to generate multiple training sets. For each training set, a decision tree is used as the base classifier. The final prediction is determined by the majority result of these multiple trees. This method can be applied not only to classification tasks but also to regression and prediction tasks. Compared with single decision tree algorithms, it delivers enhanced classification performance and is less susceptible to overfitting. In contrast, importance analysis ranks variables based on their importance by selecting the optimal variable as the classification node in the decision tree (44). The specific steps are as follows. (1) For each decision tree, select the corresponding out-of-bag (OOB) data to calculate the out-of-bag data error, denoted as err OOB1; (2) Randomly introduce noise interference to the feature X across all samples in the OOB data (by randomly altering the values of samples at feature X), subsequently recalculate the out-of-bag data error, denoted as err OOB2; and (3) Assuming there are N trees in the forest, the importance of feature X can be calculated using the following formula.


$$\begin{array}{l} \text{OOB\_store }=\;\frac{\overset{\text{N}}{\underset{\text{i}=1}{\sum }}\left(\text{erroob}2\text{i}-\text{erroob}1\text{i}\right)}{\text{N}} \end{array}$$

The reason why this value indicates a feature's importance is that if the out-of-bag data accuracy decreases significantly (i.e., err OOB2 increases) after introducing random noise, it suggests the feature exerts a substantial impact on the sample's predictive results, thereby indicating a relatively high level of importance.

#### Binary logit model

Since the reimbursement status of hospitalization expenses for the migrant population is a dichotomous variable, this paper employs a binary logit model to analyze the influencing factors of such reimbursement status using logistic regression. The basic model is as follows:


$$\text{logit}\frac{{\text{P}}_{\text{i}}}{1-{\text{p}}_{\text{i}}}=\beta_{0}+\beta_{1}{\text{X}}_{\text{i}}+\varepsilon_{\text{i}}$$

Where *P*_*i*_ denotes the probability of reimbursement of hospitalization expenses for the migrant population, β_0_ is the intercept term, *X*_*i*_ denotes other variables that would have an impact on reimbursement of hospitalization expenses, β_1_ represents the coefficient of influence of the relevant independent variable on whether or not reimbursement is made, and ε_*i*_ is the error term.

#### Linear regression model

When using logistic regression to analyze the factors influencing the hospitalization reimbursement level of the migrant population, since the reimbursement level is a continuous variable, this paper adopts a linear model to examine how each factor affects the reimbursement level. The basic model is as follows:


$$\text{Y}=\text{a}+\text{b}*{\text{X}}_{\text{i}}+\text{e}$$

Where *a* is the intercept term, *X*_*i*_ denotes the variables that would affect the level of hospitalization reimbursement, *b* represents the coefficient of the relevant independent variable on the level of hospitalization, and *e* is the error term.

#### Concentration index analysis

In public health and health-related studies, methods for measuring inequality usually include Concentration Index (CI), Gini coefficient, Oaxaca-Blinder decomposition, Lorenz curve, and extreme variance method (45, 46). Compared with other methods, the CI offers advantages: quantifying inequality direction (pro-rich/pro-poor), revealing structural inequality, decomposing factor contributions, and being unaffected by the absolute level of the indicator (47, 48). Currently, the CI is widely used to measure income-related inequalities in the health field, and has also been applied to study the equity of public health service utilization, healthcare service utilization, and health status of China's migrant population (49–52). Based on the above analysis, this study also used the CI to analyze equity in hospitalization expense reimbursement for China's migrant population and measure factor contributions. The CI formula is as follows:


$$\text{CI}=2\text{cov}({\text{y}}_{i},{\text{R}}_{i})/\mu$$

Where y_i_ represents the outcome variable of hospitalization reimbursement, μ represents the average level of the variable in the population, and R represents the fractional rank of sample *i* in the income distribution. The value of CI is in the range of (−1, 1), with CI > 0 indicating that there is a pro-rich inequality in the outcome variable, and CI < 0 indicating that there is a pro-poor inequality in the outcome variable. The larger the absolute value of CI, the more sensitive the distribution of the outcome variable is to the income level and the greater the degree of inequality.

This study adopts the CI decomposition method proposed by Wagstaff et al. (53) to decompose the factors that may affect the equity of hospitalization expense reimbursement for the migrant population, and uses the degree of contribution of different factors to equity after decomposition to rank them, to make clear the main source factors of inequity, and then to target them for control or elimination. The decomposition formula is as follows:


$$\begin{array}{l} C=\sum_{j}\left(\beta_{j}{\bar{X}}_{j}/\mu \right)C_{j}+GC_{\varepsilon }/\mu \end{array}$$

*C* is the unstandardized concentration index, β_*j*_,$\bar{X_{j}}$, and *C*_*j*_ are the regression coefficients (replaced by marginal effects), means, and concentration indices of influences *j*, respectively, $\left(\beta_{j}{\bar{X}}_{j}/\mu \right)$ indicates the magnitude of the contribution of influences *j* to inequality in hospitalization reimbursement, *GC*_ε_ is the concentration index of the residual term, and μ is the mean of the hospitalization reimbursement outcome (i.e., the dependent variable).

### Statistical analysis

**Figure 3:**
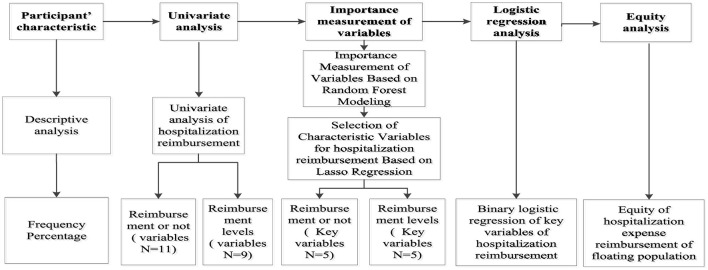
The flow chart of the study.

Data organization and analysis were conducted using Stata 22.0 and RStudio software. Categorical data were presented as counts and percentages (%), with the chi-square test applied for univariate analysis. Quantitative data conforming to a normal distribution were expressed as mean ± standard deviation (x ± s), and the *t*-test or *F*-test was used for univariate analysis. Random forest model analysis was performed in RStudio software, where variables with statistically significant differences in the univariate analysis were included in the random forest model to calculate and rank variable importance scores. LASSO analysis was employed for variable selection, and the screened variables were subjected to multivariate analysis using logistic regression. Finally, the concentration index was used to decompose hospitalization expense reimbursement, identifying the contribution of key variables to expense reimbursement. The flowchart of this study is shown in Figure 3.

## Results

### Characteristics of the respondents

Column 1 of Table 2 presents the descriptive statistics of the main variables in this study. Among the 3,186 respondents, 67.86% were under 45 years old. The educational level of the migrant population was generally low: only 23.04% had a university degree or higher, while 76.96% had an education level of high school or lower. Regarding household registration, 61.49% of the migrant population held rural household registration, and only 38.51% held urban household registration. In terms of mobility scope, the proportion of inter-city mobility within the same province was the highest, followed by inter-county mobility within the same city and inter-provincial mobility. Most of the migrant population had an income below the middle level (68.05%). For health insurance participation, the vast majority of the migrant population was covered by BMISURR (82.52%), while only 17.48% participated in BMIUE. Additionally, 62.68% of the migrant population enrolled in medical insurance in their household registration location, compared to 37.32% who enrolled in their inflow location. The health status of the migrant population was relatively good: 60.04% reported being in good health, and 25.57% reported being basically in good health.

**Table 2:** Basic characteristics of respondents.

**Variables**		**Research sample (*****N*** = **3,186)**		**Original sample (*****N*** = **152,000)**		***χ^2^***	***P***
		**1**		**2**			
		***N***	**%**	***N***	**%**		
Gender	Female	2,155	67.64	103,360	68.00	0.186	0.666
	Male	1,031	32.36	48,640	32.00		
Age	15–30	1,051	32.99	50,167	33.00	0.589	0.899
	31–45	1,011	34.87	53,178	34.99		
	46–60	652	20.46	30,424	20.02		
	61+	372	11.68	18,231	11.99		
Education	Illiterate	146	4.58	7,597	4.99	1.263	0.868
	Primary school	543	17.04	25,844	17.00		
	Junior middle school	1,115	35.00	53,201	35.00		
	Senior middle school	648	20.34	30,402	20.00		
	University/college	734	23.04	34,956	23.01		
Marriage status	Unmarried	288	9.04	13,694	9.00	0.004	0.953
	Married	2,898	90.96	138,306	91.00		
Employment	Unemployed	1,467	46.05	69,912	45.99	0.003	0.955
	Employed	1,719	53.95	82,088	54.01		
Household registration	Rural household registration	1,959	61.49	92,723	61.00	0.310	0.578
	Urban account	1,227	38.51	59,277	39.00		
Range of migration	Intercounty	893	28.03	42,567	28.00	0.361	0.835
	Intercity	1,479	46.42	69,919	45.99		
	Interprovince	814	25.55	39,514	26.01		
Reasons for migration	Family	899	28.22	42,548	27.99	0.378	0.828
	Work	2,154	67.61	103,377	68.01		
	Others	133	4.17	6,075	4.00		
Household income ranking	Lowest ( ≤ percentile 20)	928	29.13	44,088	29.01	0.491	0.974
	Lower (percentile 20–39)	657	20.62	31,925	21.00		
	Middle (percentile 40–59)	583	18.30	27,362	18.00		
	Higher (percentile 60–79)	546	17.14	25,833	16.99		
	Highest (≥percentile 80)	472	14.81	22,792	14.98		
Type of health insurance	BMISURR	2,629	82.52	125,983	83.00	0.515	0.473
	BMIUE	577	17.48	25,804	17.00		
Health insurance location	Household registration	1,997	62.68	94,846	63.00	0.137	0.712
	Place of inflow	1,189	37.32	55,703	37.00		
Health status	Unhealthy	522	16.38	15,655	10.30	678.705	< 0.001
	Basically healthy	751	25.57	16,854	11.10		
	Healthy	1,913	60.04	119,491	78.60		
Total medical expenditure(USD)	≤ 1,221	1,051	32.99	1,694	34.93	3.277	0.194
	1,221–2,442	1,160	36.41	1,704	35.13		
	≥2,442	975	30.60	1,452	29.94		
Disease condition	No	2,893	90.80	138,327	91.00	0.154	0.695
	Yes	293	9.20	13,673	9.00		

The sample size of the insured location and total medical expenditure in the original database was included in the analysis based on the actual valid quantity.

To evaluate the representativeness of the study sample, we also conducted a comparative descriptive statistical analysis of the original sample from the 2018 CMDS database (Column 2 of Table 2) and compared its results with those of the sample included in this study. The analysis showed that the difference between the final and original samples in the distribution of health levels was statistically significant, but the differences between these two samples in other characteristics failed statistical tests. This suggests that the sample included in the study has relatively good representativeness. As for the difference in health status distribution between the two samples, it may be attributed to the fact that this study focuses on analyzing the hospitalization behavior and hospitalization expenses of the migrant population. Since this specific population may have poorer health status, the proportion of respondents in an unhealthy state in the study sample is higher than that in the original sample, while the proportion of those in a healthy state is lower.

### Current situation of reimbursement of hospitalization expenses for the migrant population

**Figure 4:**
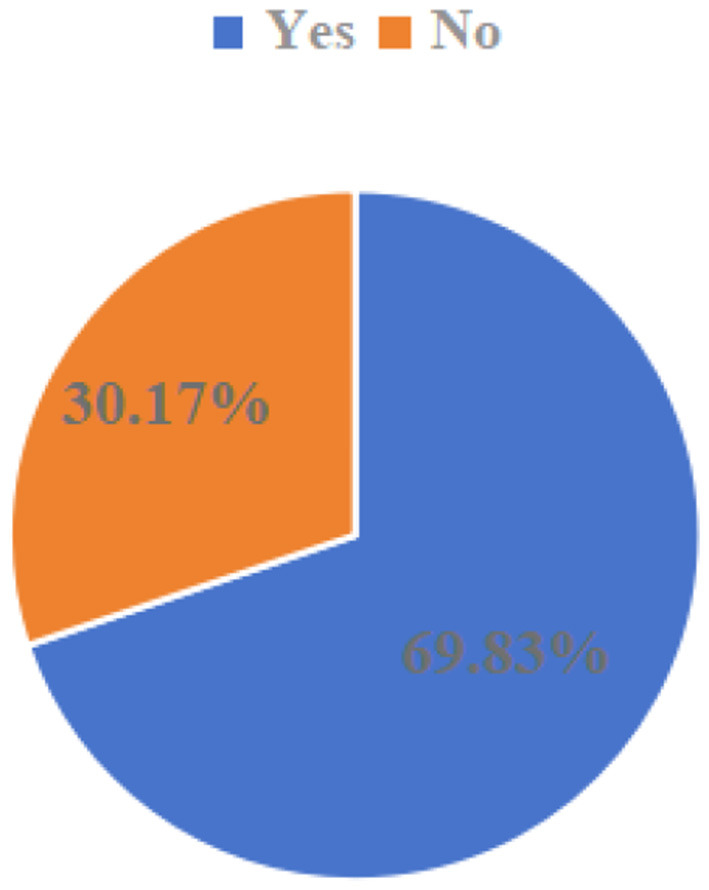
Hospitalization cost reimbursement options for migrant populations.

To examine the current status of hospitalization expense reimbursement among the migrant population, a comprehensive analysis was conducted across multiple dimensions, including reimbursement status (whether reimbursement is obtained), reimbursement location, reimbursement method, and reimbursement level. First, regarding the migrant population's choice of hospitalization expense reimbursement: as shown in Figure 4, when incurring hospitalization expenses, 69.83% of the migrant population opted to claim reimbursement, while 30.17% did not seek reimbursement.

**Figure 5:**
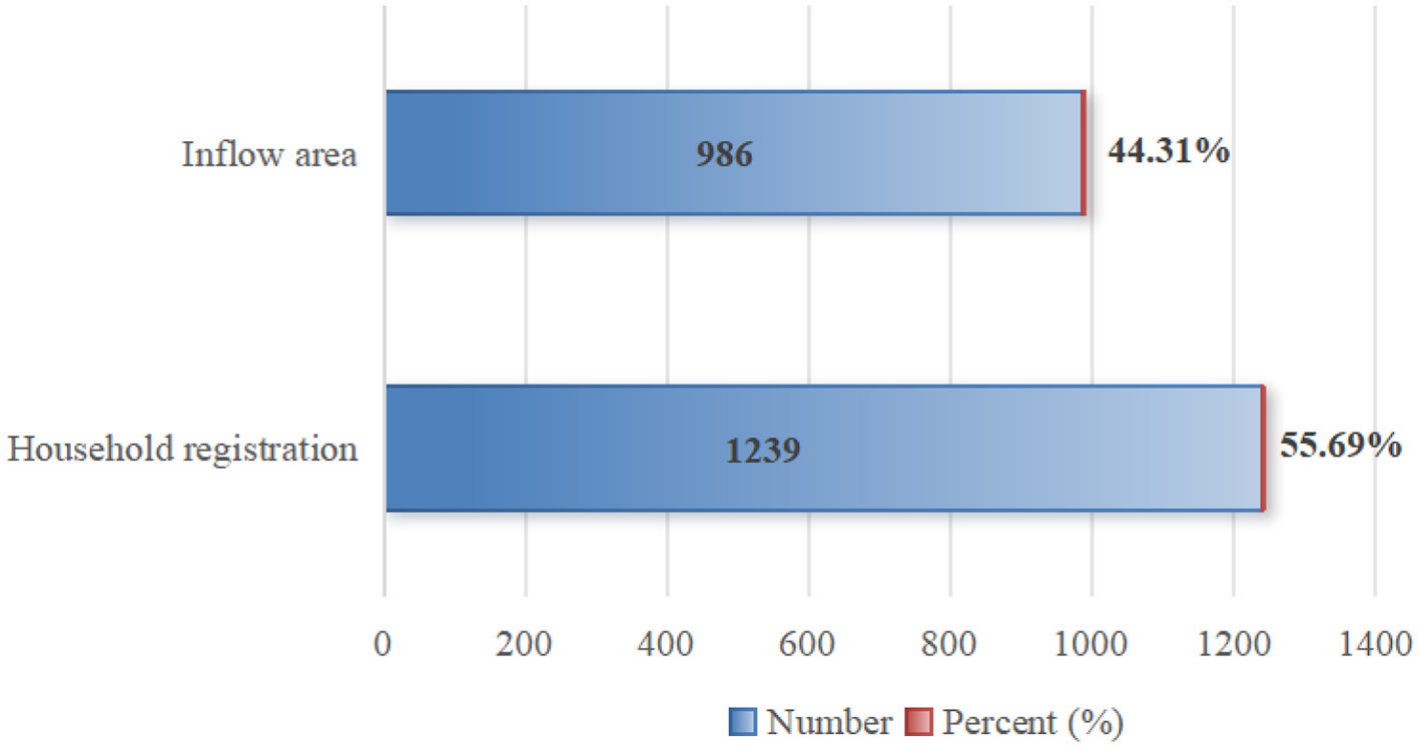
Location of reimbursement of hospitalization expenses for migrant populations.

**Figure 6:**
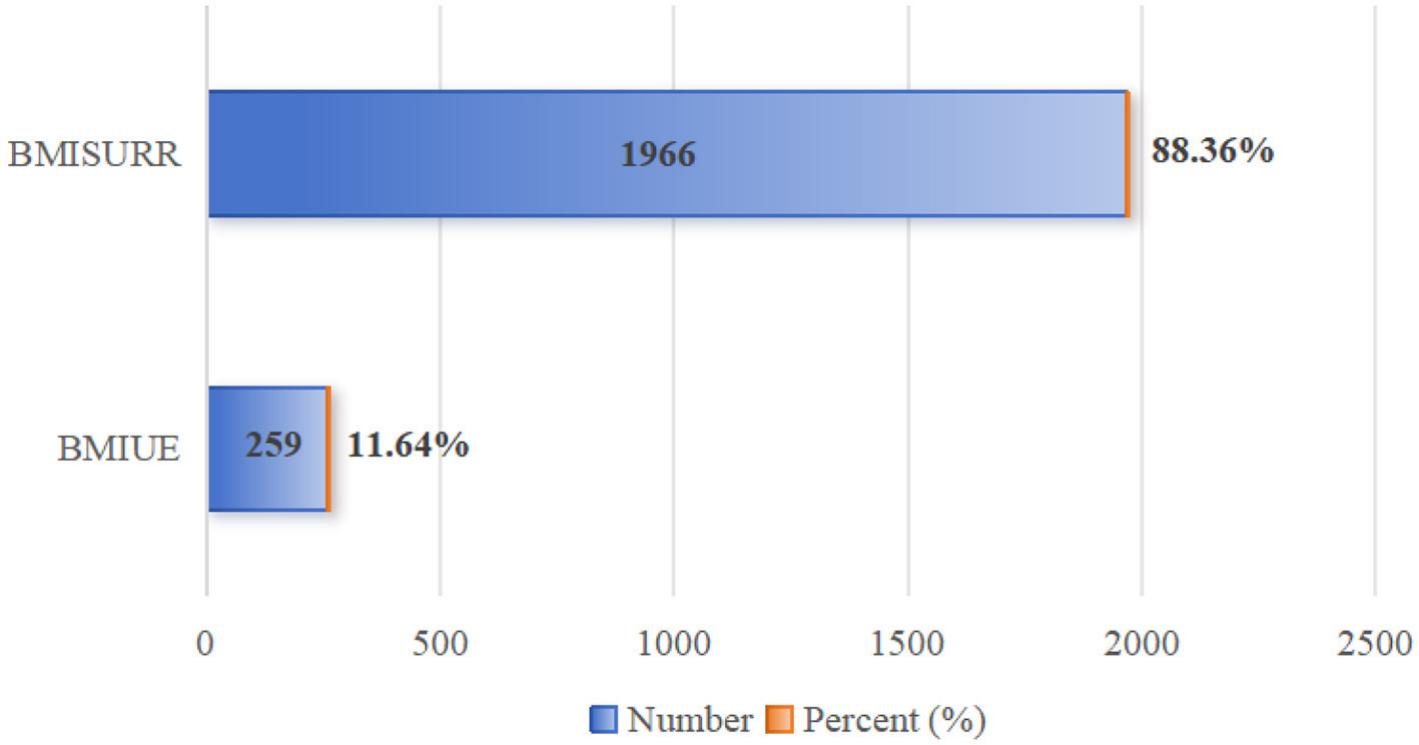
Types of reimbursement insurance for hospitalization expenses of migrant population.

Second, regarding the location and method of hospitalization expense reimbursement among the migrant population: as shown in Figure 5, 55.69% of the migrant population reimbursed their hospitalization expenses in their place of domicile, while 44.31% did so in their place of inflow—meaning the proportion of reimbursement in the place of domicile was more than twice that in the place of inflow. In terms of reimbursement methods (Figure 6), 1,966 individuals opted for reimbursement through BMISURR, accounting for 88.36%, while 259 chose BMIUE, representing 11.64%.

**Figure 7:**
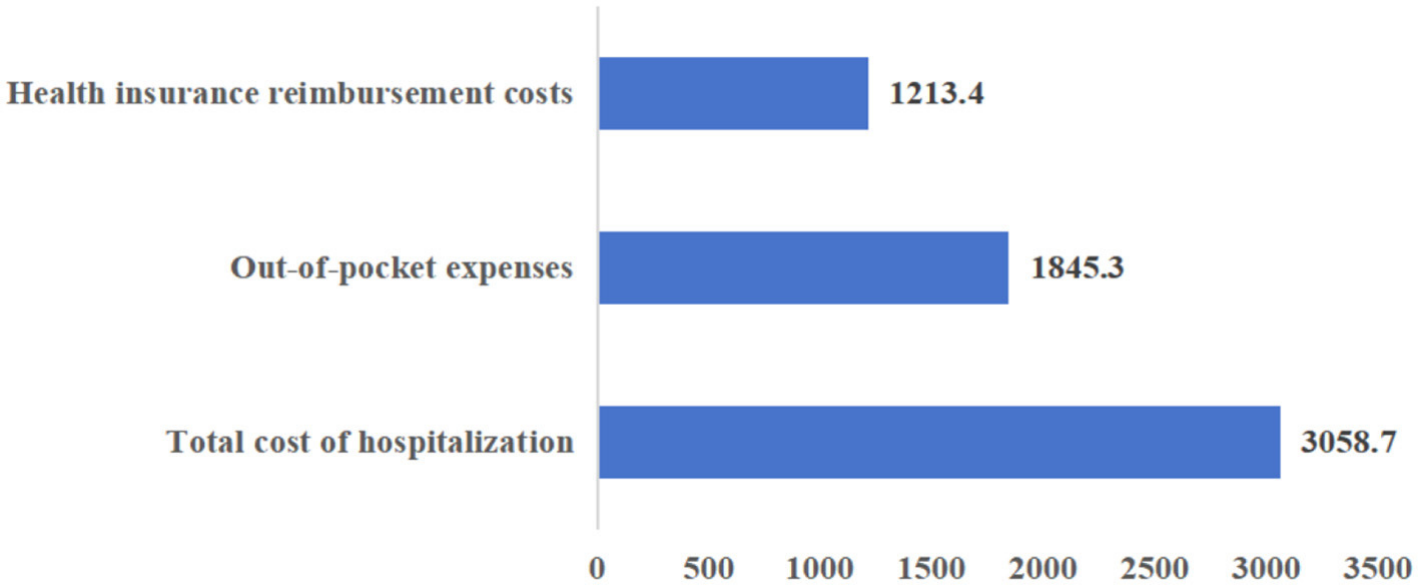
Analysis of the composition of hospitalization expenses of migrant population.

**Figure 8:**
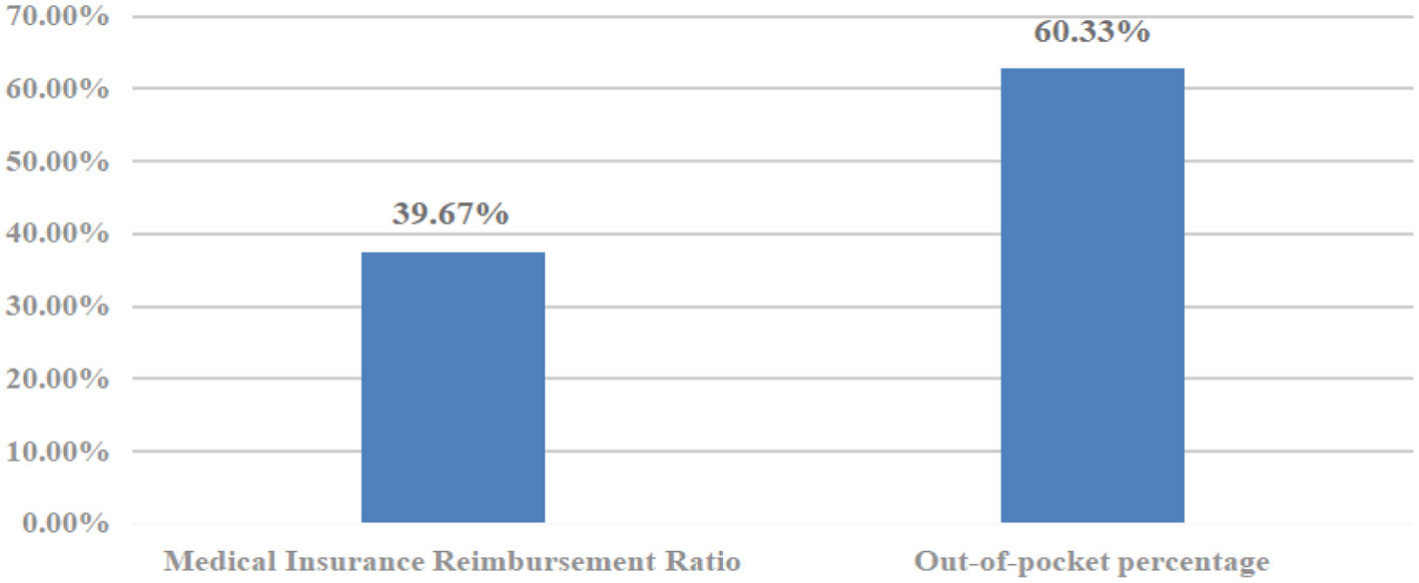
Payment structure of hospitalization expenses of migrant population.

Finally, the level of hospitalization expense reimbursement among the migrant population is analyzed in Figure 7. The average total hospitalization expense stands at 3,058.7 USD, with 1,213.4 USD covered by health insurance reimbursement and 1,845.3 USD paid out-of-pocket by the migrant population themselves. As shown in Figure 8, the proportion of hospitalization expenses reimbursed by health insurance is 39.67%, while the proportion borne by the migrant population through self-payment is 60.33%.

### Univariate analysis of hospitalization expense reimbursement for the migrant population

Table 3 presents the results of the univariate analysis with hospitalization expense reimbursement status and reimbursement amount as the dependent variables. For hospitalization expense reimbursement status, statistically significant differences were observed in the migrant population's choice of reimbursement based on gender, age, educational level, marital status, household registration type, mobility range, reason for mobility, income level, insurance enrollment location, insurance type, and health status. Regarding the reimbursement level, statistically significant differences were found in the proportion of hospitalization expenses reimbursed among the migrant population across variables including age, marital status, occupation, mobility range, reason for mobility, insurance type, health status, total medical expenditure, and type of illness.

**Table 3:** Reimbursement of hospitalization expenses of migrant population under different characteristics.

**Variables**		**Reimbursement or not**						**Level of reimbursement**			
		**Yes**		**No**		*χ^2^*	***P***	$\bar{x}$	**S**	***t/F***	***P***
		***n***	**%**	***n***	**%**						
Gender	Female	1,472	46.20	683	21.44	7.405	0.007	0.498	0.005	−1.714	0.957
	Male	753	23.63	278	8.73			0.513	0.007		
Age	15–30	659	20.68	392	12.30	63.745	< 0.001	0.296	0.230	111.96	< 0.001
	31–45	770	24.17	341	10.70			0.507	0.231		
	46–60	487	15.29	165	5.18			0.605	0.227		
	61+	309	9.70	63	1.98			0.719	0.224		
Education	Illiterate	103	3.23	43	1.35	30.465	< 0.001	0.519	0.226	0.24	0.915
	Primary school	384	12.05	159	4.99			0.502	0.231		
	Junior middle school	727	22.82	388	12.18			0.500	0.229		
	Senior middle school	445	13.97	203	6.37			0.502	0.235		
	University/college	566	17.77	168	5.27			0.506	0.243		
Marriage Status	Unmarried	188	5.90	100	3.14	3.124	0.077	0.544	0.014	3.085	0.001
	Married	2,037	63.94	861	27.02			0.499	0.004		
Employment	Unemployed	1,019	31.98	448	14.06	0.182	0.670	0.479	0.032	18.266	< 0.001
	Employed	1,206	37.85	513	16.10			0.495	0.016		
Household registration	Rural household registration	1,297	40.71	662	20.78	31.811	< 0.001	0.503	0.005	0.010	0.496
	Urban account	928	29.13	299	9.38			0.503	0.007		
Range of migration	Intercounty	669	21.00	224	7.03	20.134	< 0.001	0.481	0.031	127.701	< 0.001
	Intercity	1,027	32.23	452	14.19			0.496	0.018		
	Interprovince	529	16.60	285	8.95			0.482	0.028		
Reasons for migration	Family	643	20.18	256	8.04	16.849	< 0.001	0.450	0.005	11,823.977	< 0.001
	Work	1,470	46.14	684	21.47			0.500	0.010		
	Others	112	3.52	21	0.66			0.548	0.019		
Household income ranking	Lowest (≤ percentile 20)	636	19.96	292	9.17	8.717	0.069	0.503	0.236	0.67	0.612
	Lower (percentile 20–39)	435	13.65	222	6.97			0.501	0.228		
	Middle (percentile 40–59)	422	13.25	161	5.05			0.508	0.229		
	Higher (percentile 60–79)	393	12.34	153	4.80			0.512	0.237		
	Highest (≥percentile 80)	339	10.64	133	4.17			0.489	0.235		
Type of health insurance	BMISURR	1,966	61.71	663	20.81	174.528	< 0.001	0.097	0.255	−20.51	< 0.001
	BMIUE	259	8.13	298	9.35			0.569	0.275		
Health insurance location	Household registration	1,239	39.00	758	24.0	154.301	< 0.001	0.503	0.005	0.044	0.484
	Place of inflow	986	31.00	203	6.00			0.502	0.007		
Health status	Unhealthy	413	12.96	109	3.42	41.198	< 0.001	0.565	0.328	916.76	< 0.001
	Basically healthy	553	17.36	198	6.21			0.657	0.377		
	Healthy	1,259	39.52	654	20.53			0.698	0.325		
Total medical expenditure (USD)	≤ 1,221	731	32.85	320	33.30	0.106	0.948	0.031	0.259	1,467.74	< 0.001
	1,221–2,442	814	36.58	346	36.00			0.335	0.249		
	≥2,442	680	30.56	295	30.70			0.653	0.266		
Disease condition	No	2,024	90.97	869	90.43	0.234	0.629	0.490	0.000	−3.81e+15	< 0.001
	Yes	201	9.03	92	9.57			0.510	0.000		

### Importance measurement of factors influencing hospitalization expense reimbursement for migrant population

#### Random forest data selection

**Figure 9:**
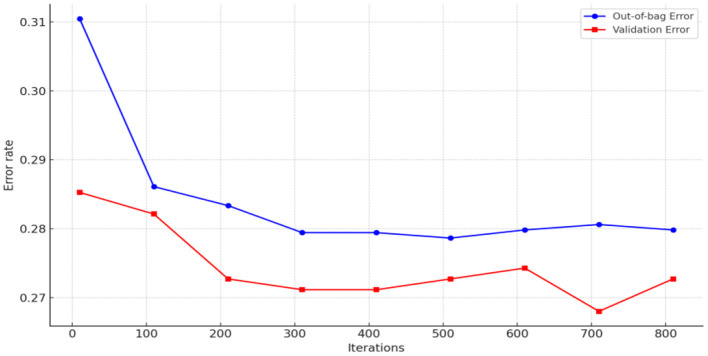
Determining the number of model decision trees (whether hospitalization expenses are reimbursed or not).

**Figure 10:**
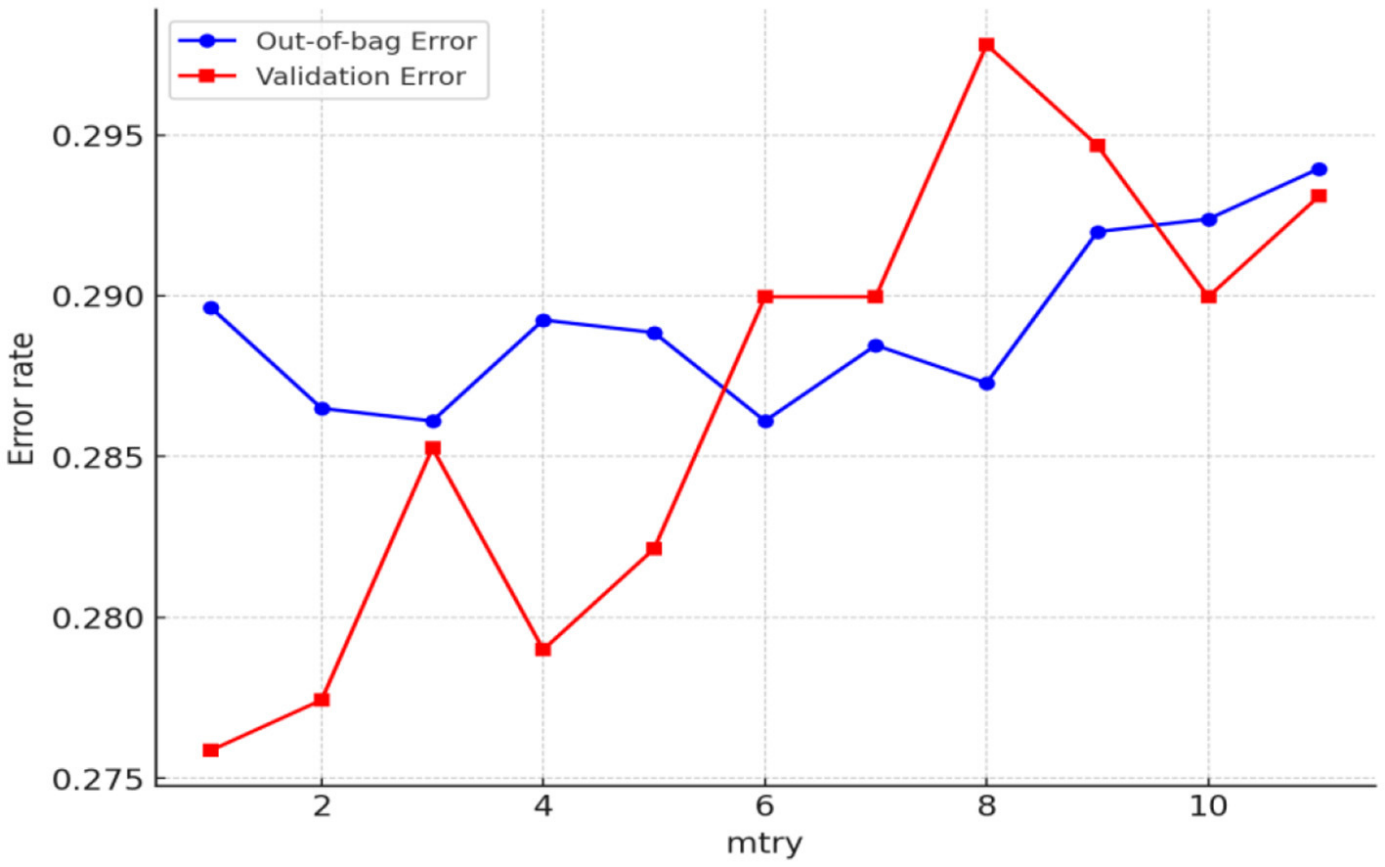
Determining the number of model feature trees (whether hospitalization expenses are reimbursed or not).

To measure the importance of the factors affecting the hospitalization expense reimbursement for the migrant population in China, a random forest model for hospitalization expense reimbursement was first established in this study, with “whether hospitalization expenses are reimbursed or not” as the dependent variable and factors affecting the migrant population's hospitalization expenses as independent variables. The results are presented in Figures 9, 10. In this study, samples were randomly divided into a training set (80%) and a validation set (20%). Two key parameters are involved in building the random forest model: one is the number of decision trees in the forest, and the other is the number of variables selected randomly for splitting at each node during the generation of each decision tree. As shown in Figure 9, the model tends to stabilize when the number of decision trees reaches 400. For prudence, the number of iterations was set to 500 in this study. After model tuning, Figure 10 indicates that the number of variables used for splitting minimizes the mean square error within the range of 2–10.

The random forest was further optimized through random search to confirm the optimal parameter combination. Based on the entry in the range of (2–10) and three in the range of (0–500) obtained from the above two graphs, the maximum depth max_depth range of the tree (2–20) and the minimum range of the leaf nodes min_samples_leaf (1–10) are also added to perform the random combinations and get the optimal parameter of the random forest by minimizing the MSE value combination, as shown in Table 4.

**Table 4:** Optimal parameter combinations for random forest analysis (probability of hospitalization reimbursement).

**Items**		**Parameter**
max_depth	Maximum depth of a tree in a random forest	4
min_samples_leaf	Minimum sample size of leaf nodes in a random forest	8
min_samples_split (mtry)	Minimum number of samples required to split internal nodes	7
n_estimators (ntree)	Number of trees in a randomized forest	214
random_state	Random seed	Default randomized value(42)

**Figure 11:**
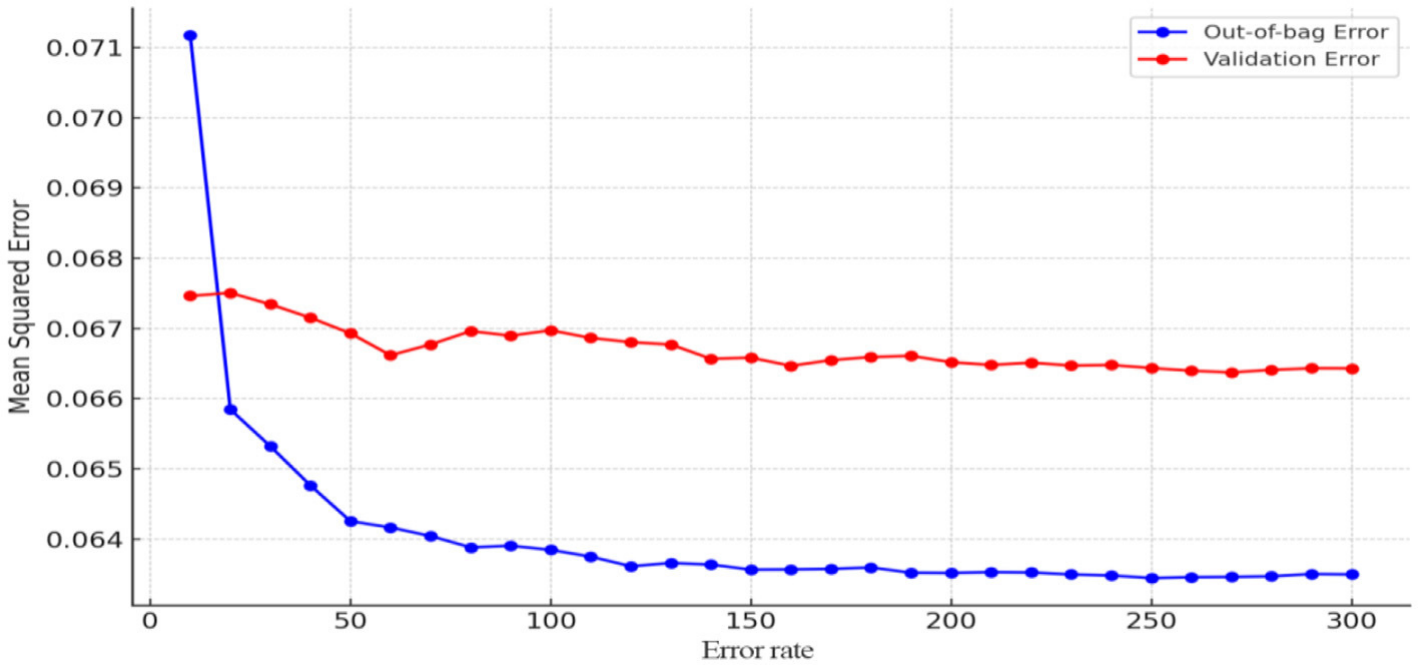
Determining the number of model decision trees (hospitalization expense reimbursement level).

**Figure 12:**
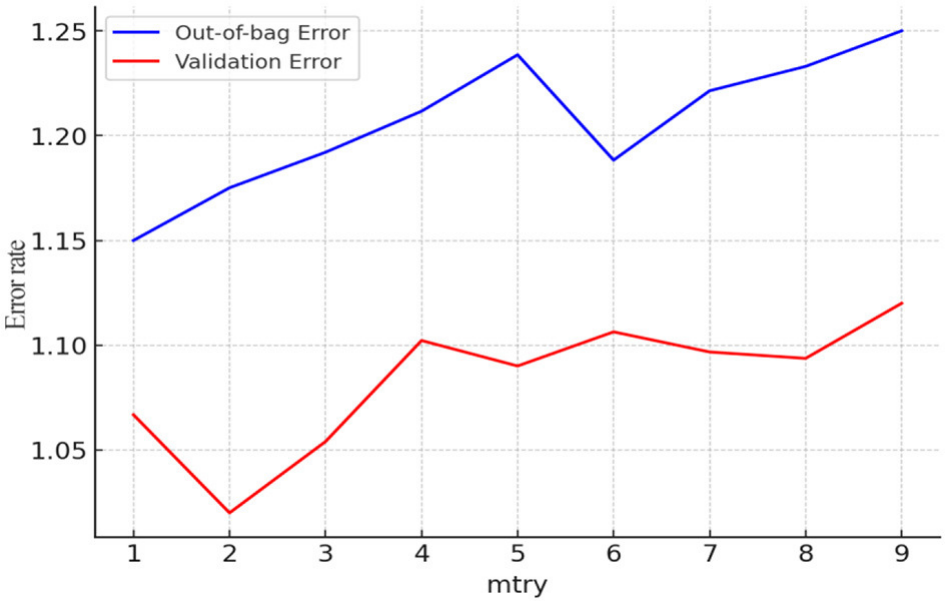
Determining the number of model feature trees (hospitalization expense reimbursement level).

Additionally, to assess the importance of factors influencing the hospitalization expense reimbursement level among China's migrant population, this study established a random forest model for the reimbursement level, with the hospitalization expense reimbursement level as the dependent variable and the factors affecting this level as independent variables. The results are presented in Figures 11, 12. As shown in Figure 11, the model tends to stabilize when the number of decision trees reaches 150. For prudence, the number of iterations was set to 250 in this study. After model tuning, Figure 12 indicates that the number of splitting variables minimizes the mean squared error (MSE) within the range of 2–9.

The random forest was further optimized through random search to confirm the optimal parameter combination. Based on the entry in the range of (2–9) and three in the range of (0–300) obtained from the above two graphs, the maximum depth max_depth range of the tree (2–20) and the minimum range of the leaf nodes min_samples_leaf (1–10) are also added to perform the random combinations and obtain the optimal parameter combinations of the random forest by minimizing the MSE values, as shown in the Table 5.

**Table 5:** Optimal parameter combinations for random forest analysis (level of reimbursement of hospitalization expenses).

**Items**		**Parameter**
max_depth	Maximum depth of a tree in a random forest	2
min_samples_leaf	Minimum sample size of leaf nodes in a random forest	9
min_samples_split (mtry)	Minimum number of samples required to split internal nodes	6
n_estimators (ntree)	Number of trees in a randomized forest	133
random_state	Random seed	Default randomized value (42)

**Figure 13:**
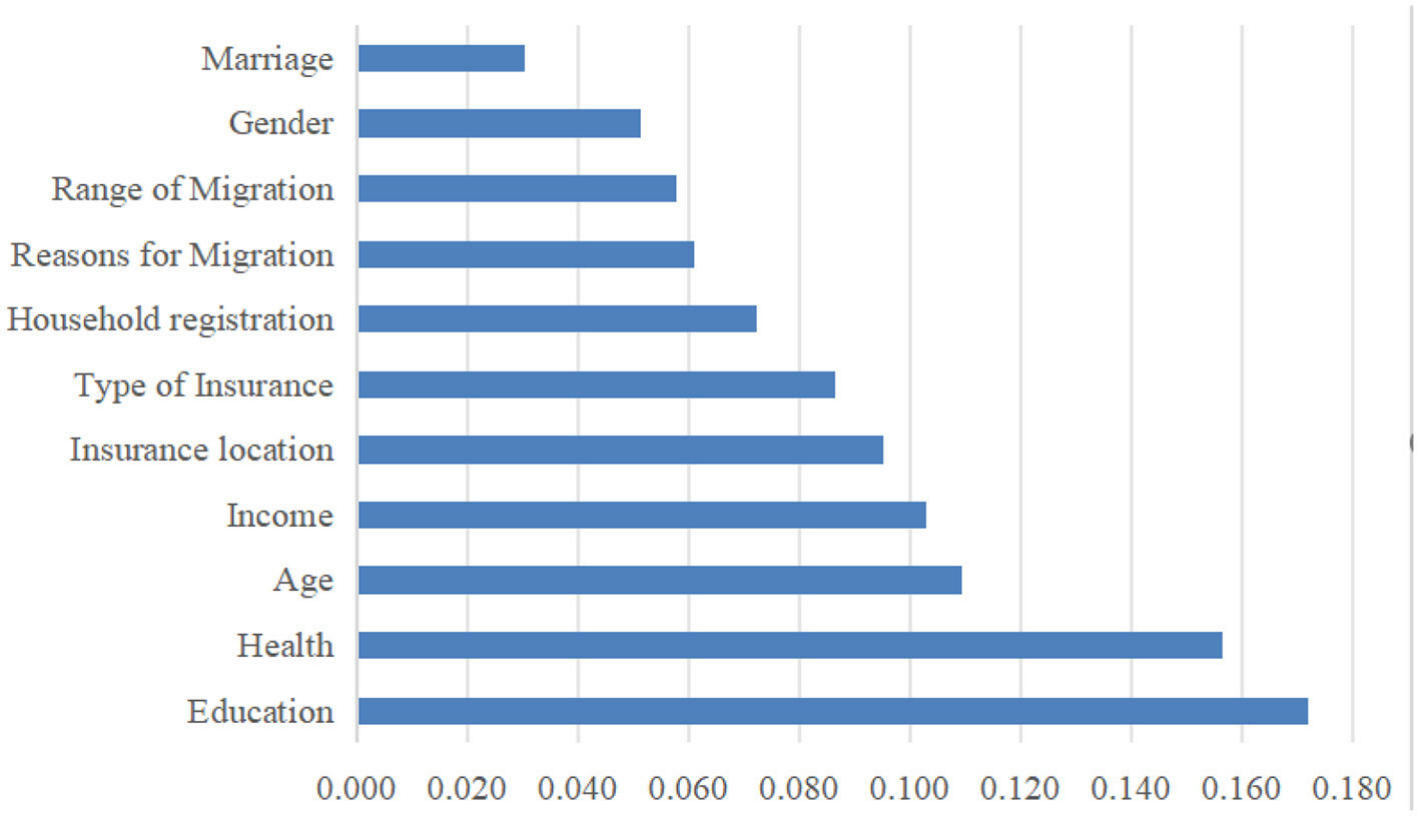
Importance ranking of factors influencing whether or not hospitalization costs are reimbursed for the mobile population.

**Figure 14:**
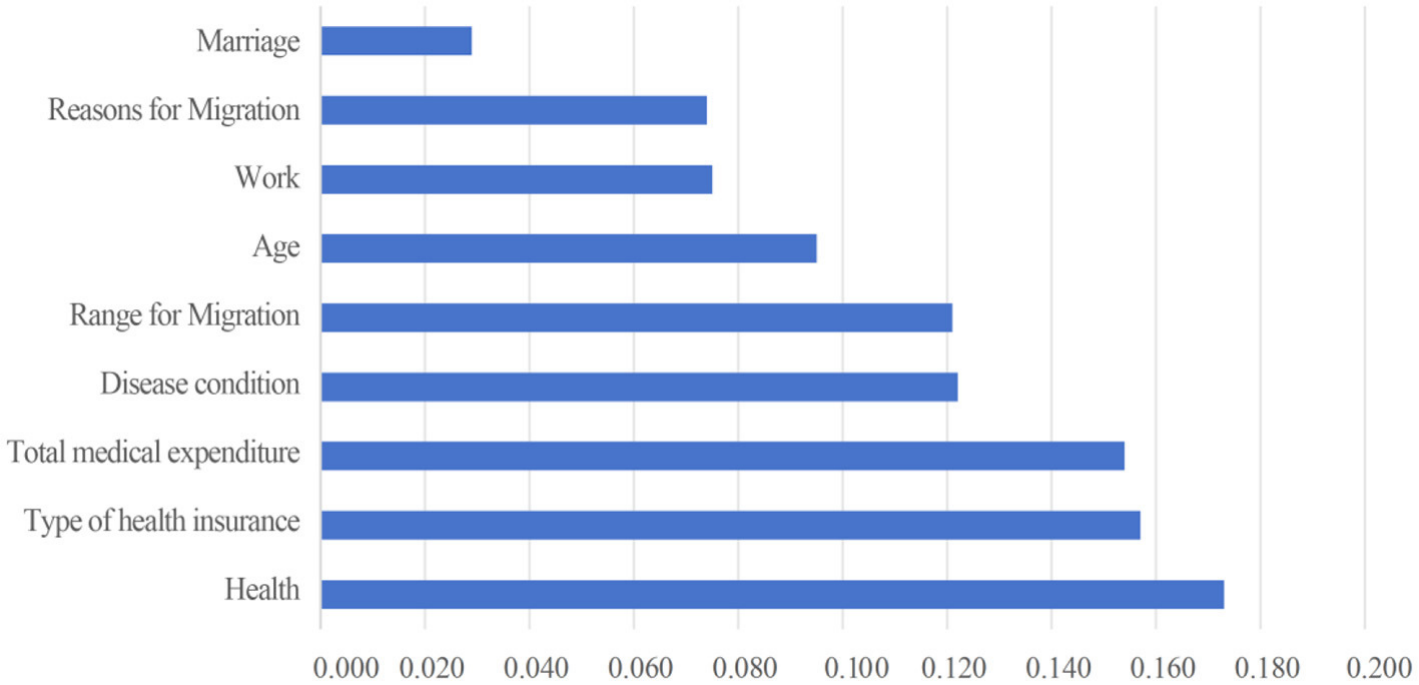
Importance ranking of factors influencing the level of reimbursement of hospitalization costs for the mobile population.

To rank the importance of factors influencing hospitalization expense reimbursement (both in terms of reimbursement status and reimbursement level), variables with statistically significant differences in the univariate analysis were included in the random forest model, with “whether hospitalization expenses are reimbursed” and “hospitalization expense reimbursement level” serving as the dependent variables, respectively. The results were generated using the RandomForest package in RStudio. %Inc MSE (Increase in Mean Squared Error) represents the average reduction in model precision; a larger %Inc MSE indicates a higher importance of the variable among the influencing factors (54). Figures 13, 14 present the importance assessment results for factors influencing hospitalization expense reimbursement among the migrant population. For the factors affecting whether the migrant population receives hospitalization expense reimbursement, their importance in descending order is: educational level, health status, age, income level, local insurance enrollment status, insurance type, household registration type, reason for mobility, mobility range, gender, and marital status. For the factors affecting the level of hospitalization expense reimbursement, their importance in descending order is: health status, insurance type, total medical expenditure, illness status, mobility range, age, occupation, reason for mobility, and marital status.

#### Characteristic variable screening

**Figure 15:**
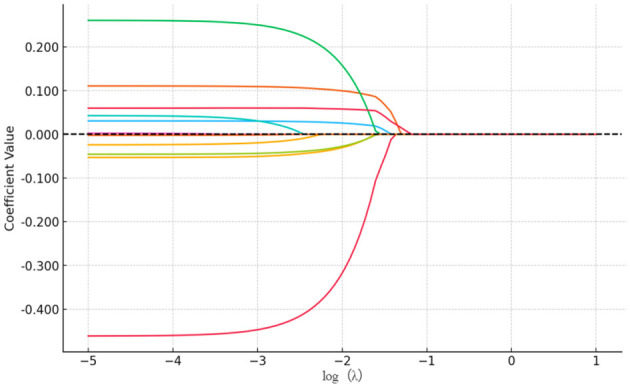
LASSO variable screening map. A total of 11 variables were included in this study and the figure shows 11 lines of different shades of color. Each curve represents the trajectory of the coefficient of each independent variable, with the vertical coordinate being the value of the coefficient and the lower horizontal coordinate being the value of log (λ).

**Figure 16:**
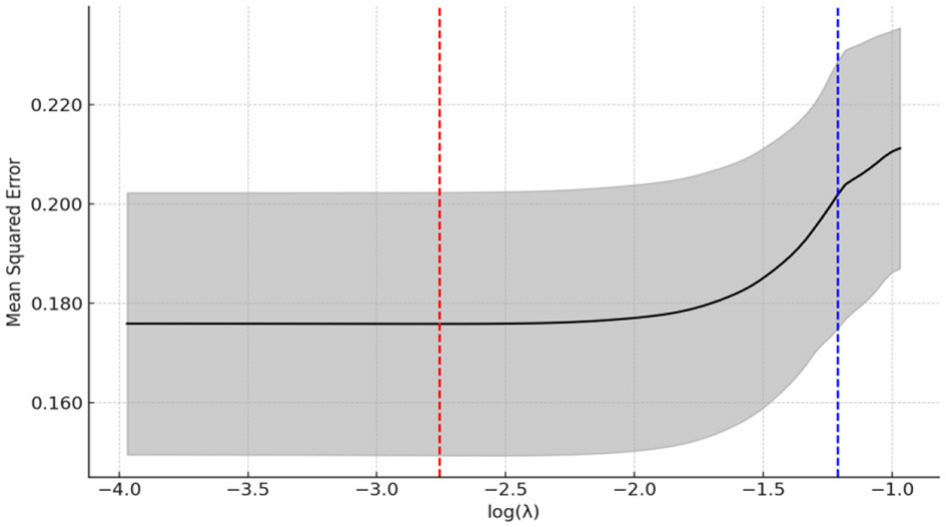
LASSO regression selection for optimal parameters. The figure screens the number of feature variables based on the minimum criterion by plotting dashed lines at lambda.min (left dashed line) and lambda.lse (right dashed line), respectively, with the optimal parameter position being the value of λ at the minimum model error, i.e., the dashed line on the left.

The LASSO regression method can achieve feature selection and dimensionality reduction through L1 regularization, which sets the coefficients of unimportant features to zero. This approach can screen variables to avoid multicollinearity and overfitting. Compared with traditional stepwise regression, it processes all independent variables simultaneously, thereby enhancing model stability. In this study, the inclusion of a large number of independent variables increases the risk of multicollinearity (55, 56). Thus, the LASSO regression model was employed for variable selection, and the selected important variables were further analyzed using logistic regression. The LASSO analysis was performed using the glmnet package in R, with 100 iterations. The optimal λ (lambda) value was determined via 10-fold cross-validation. Figures 15, 16 present the results of selecting important variables influencing whether the migrant population receives hospitalization expense reimbursement. Calculations showed that the cross-validation error is minimized when λ = −2.757, making this the optimal regularization parameter, corresponding to five influencing factors. Therefore, the top five variables (educational level, health status, age, income level, and insurance enrollment location) were included in the multiple stepwise regression analysis.

**Figure 17:**
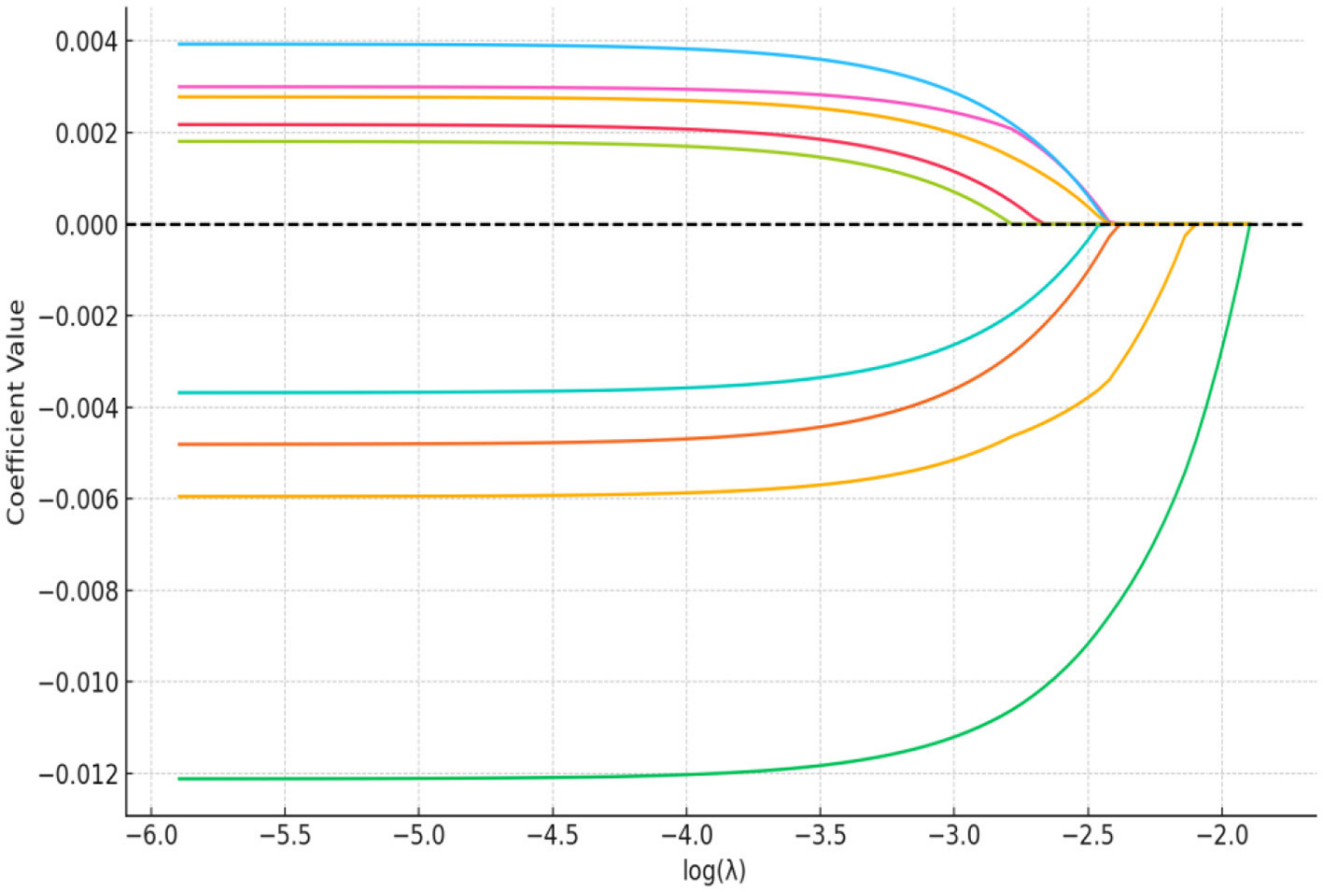
LASSO variable screening map. A total of nine variables were included in this study and the figure shows nine lines of different shades of color. Each curve represents the trajectory of the coefficient of each independent variable, with the vertical coordinate being the value of the coefficient and the lower horizontal coordinate being the value of log (λ).

**Figure 18:**
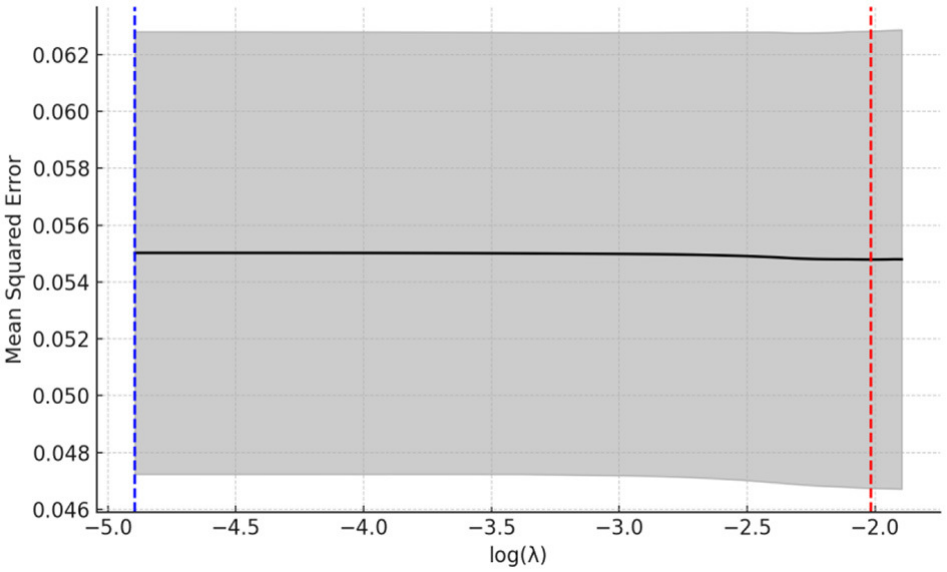
LASSO regression selection for optimal parameters. The figure screens the number of feature variables based on the minimum criterion by plotting dashed lines at lambda.min (left dashed line) and lambda.lse (right dashed line), respectively, with the optimal parameter position being the value of λ at the minimum model error, i.e., the dashed line on the left.

The results of the analysis of the selection of important variables for the level of hospitalization expense reimbursement among the migrant population are shown in Figures 17, 18. By calculation, the cross-validation error is minimized when the value of lambda (λ) is −4.895, which is the optimal regularization parameter, and the number of influencing factors corresponding to it is five. Accordingly, the top five variables, including health status, insurance type, total medical expenditure, illness status, and mobility range, were included in the multiple stepwise regression analysis.

### Regression analysis of factors affecting hospitalization expense reimbursement for the migrant population

#### Regression analysis of factors influencing whether hospitalization expenses are reimbursed or not

Binary logistic regression analysis was conducted with hospitalization expense reimbursement status as the dependent variable and the top 5 variables screened by the random forest model as independent variables, and the results are presented in Table 6. Migrant individuals with a high school education (OR = 1.688, 95% CI: 1.068, 2.667) and those with a college education or higher (OR = 2.015, 95% CI: 1.249, 3.251) had a higher probability of receiving hospitalization expense reimbursement. Compared to those in an unhealthy state, migrant individuals in a healthy state were less likely to be reimbursed for hospitalization expenses (OR = 0.562, 95% CI: 0.416, 1.759). Older individuals in the migrant population had a higher likelihood of obtaining hospitalization expense reimbursement, with the highest Predisposing observed among those aged over 61 years (OR = 3.176, 95% CI: 2.159, 4.672). Compared to the lowest income group, the probability of hospitalization expense reimbursement for the migrant population increased gradually with rising income levels, showing a linear relationship between the two. The highest probability of reimbursement was found among those in the highest income range (OR = 2.826, 95% CI: 1.994, 4.006). In comparison to those enrolled in health insurance at their place of domicile, migrant individuals enrolled in health insurance at their place of inflow were more likely to receive hospitalization expense reimbursement (OR = 2.735, 95% CI: 2.251, 3.323), indicating that local health insurance enrollment expands the reimbursement options available to the migrant population.

**Table 6:** Binary logistic regression analysis affecting the reimbursement or non-reimbursement of hospitalization expenses for the migrant population.

**Variables**		**β**	***P***	**OR**	**95%CI**
Education	Illiterate (reference)				
	Primary school	0.371	0.100	1.449	0.932, 2.254
	Junior middle school	0.455	0.040	1.576	1.021, 2.431
	Senior middle school	0.524	0.025	1.688	1.068, 2.667
	University/college	0.701	0.004	2.015	1.249, 3.251
Health	Unhealthy (reference)				
	Basically healthy	−0.137	0.380	0.872	0.643, 1.184
	Healthy	−0.576	0.000	0.562	0.416, 1.759
Age	15–30 (reference)				
	31–45	0.243	0.018	1.275	1.043, 1.558
	46–60	0.619	0.000	1.858	1.414, 2.442
	61–	1.156	0.000	3.176	2.159, 4.672
Income	Lowest (< percentile 20) (reference)				
	Lower (percentile 20–39)	0.009	0.932	1.009	0.804, 1.269
	Middle (percentile 40–59)	0.252	0.044	1.287	1.006, 1.647
	Higher (percentile 60–79)	0.267	0.047	1.306	1.004, 1.699
	Highest (≥percentile 80)	1.039	0.000	2.826	1.994, 4.006
Health insurance location	Household registration (reference)				
	Place of inflow	1.006	0.000	2.735	2.251, 3.323

For the regression analysis of factors influencing the hospitalization expense reimbursement level, multiple regression analysis was conducted with the hospitalization expense reimbursement level as the dependent variable and the top 5 most important variables screened by the random forest model as independent variables. The results indicated that health status, insurance type, total medical expenditure, illness status, and mobility range were the main factors affecting the hospitalization expense reimbursement level of the migrant population (*P* < 0.001), as presented in Table 7.

**Table 7:** Multiple regression analysis affecting the level of reimbursement of hospitalization costs for the migrant population.

**Variables**	**β**	**SE**	***t*-value**	***P***	**95%CI**
Constant	0.4992	0.0022	223.36	< 0.001	0.4948, 0.5036
Health	−0.0196	0.0005	−41.55	< 0.001	−0.0206, −0.0187
Type of health insurance	0.0310	0.0013	24.74	< 0.001	0.0285, 0.0334
Total medical expenditure (USD)	0.0398	0.0004	88.74	< 0.001	0.0390, 0.0407
Disease condition	−0.0358	0.0012	−28.93	< 0.001	−0.0382, −0.0334
Range of migration	0.0602	0.0013	46.36	< 0.001	0.0576, 0.0628

### Equity analysis of hospitalization expense reimbursement for migrant population

#### Equity evaluation of the probability of hospitalization expense reimbursement

**Figure 19:**
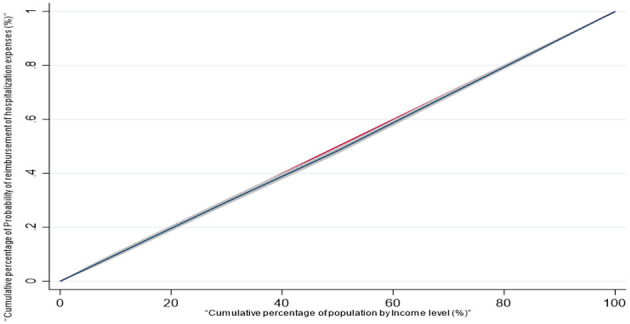
Concentration curve of the probability of reimbursement of hospitalization costs for the mobile population.

For the CI regarding whether the migrant population receives hospitalization expense reimbursement, the calculated CI is 0.014. Since the concentration index is >0, and the concentration curve exhibits a concave trend below the absolute equality line (Figure 19), this indicates that the distribution of the probability of hospitalization expense reimbursement is more skewed toward the population with a higher income level.

#### Decomposition of the contribution of key factors to the equity of the probability of reimbursement of hospitalization expenses

Taking the five key factors selected by LASSO as independent variables and “whether the migrant population is reimbursed for hospitalization expenses” as the dependent variable, this study further explored the contribution of different factors to the equity of the reimbursement probability. The analysis results are presented in Table 8. From the concentration index decomposition results, the concentration indices of inequalities in hospitalization reimbursement probability associated with educational level, health status, age, income level, and insurance enrollment location are all positive. This indicates that these five factors collectively contribute to the inequality in hospitalization expense reimbursement among the migrant population. In descending order of their contribution magnitude to the inequality, the factors are: educational level (42.3%), income level (34.1%), health status (12.4%), age (8.2%), and insurance enrollment location (3.0%).

**Table 8:** Concentration index decomposition of the probability of reimbursement of hospitalization costs for the mobile population.

**Variables**	**Elasticity coefficient**	**CI**	**Contribution**	**Contribution rate (%)**
Education	0.218	0.402	0.720	42.3
Health	0.073	0.128	0.210	12.4
Age	0.045	0.085	0.140	8.2
Income	0.185	0.341	0.580	34.1
Health insurance location	0.021	0.044	0.050	3.0

#### Equity evaluation of hospitalization expense reimbursement level

**Figure 20:**
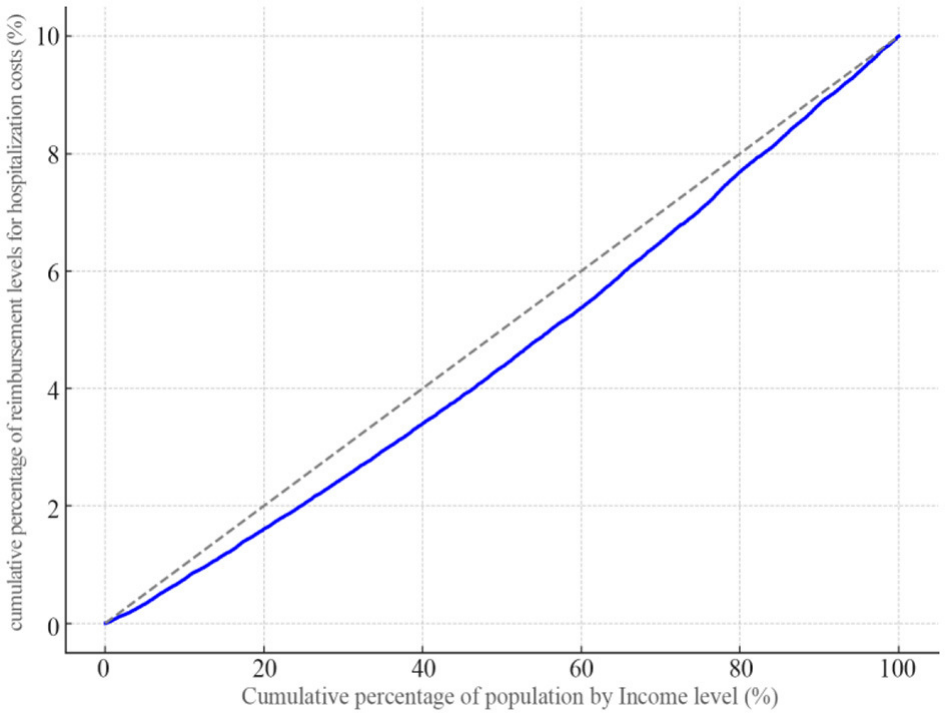
Concentration curve of the level of reimbursement of hospitalization costs for the mobile population.

For the CI of the hospitalization expense reimbursement level among the migrant population, the calculated CI is 0.082, which is >0. The concentration curve presents a concave trend and lies below the absolute equality line (Figure 20), indicating that the distribution of the hospitalization expense reimbursement level is more inclined toward the population with a higher income level.

#### Decomposition of the contribution of key factors to the equity of hospitalization expense reimbursement level

With the five key factors selected by LASSO as independent variables and the hospitalization expense reimbursement level of the migrant population as the dependent variable, this study further explored the contribution of different factors to the equity of the reimbursement level, and the analysis results are shown in Table 9. From the concentration index decomposition results, the concentration indices for the contribution of health status, health insurance type, insurance enrollment location, mobility range, and occupation to the inequality in hospitalization expense reimbursement among the migrant population are all positive. This indicates that these five factors collectively contribute to the inequality in the reimbursement level. Ranked in descending order of their contribution to the inequality, the factors are: health status (58.12%), mobility location (21.74%), total medical expenditure (9.35%), health insurance type (9.28%), and illness status (1.51%).

**Table 9:** Concentration index decomposition of the level of reimbursement of hospitalization costs for the mobile population.

**Variables**	**Elasticity coefficient**	**CI**	**Contribution**	**Contribution rate (%)**
Health	0.0547	0.0312	0.00171	58.12
Type of health insurance	0.7581	0.0004	0.00027	9.28
Range of migration	0.0126	0.0506	0.00064	21.74
Total medical expenditure (USD)	0.1014	0.0027	0.00028	9.35
Disease condition	0.0023	0.0189	0.00004	1.51

## Discussion

This study aims to analyze the key influencing factors of hospitalization expense reimbursement and its equity among the China's migrant population. This research evidence from China can not only enrich the existing theoretical research on the health service utilization by the migrant population, but also provide practical support for optimizing their expense reimbursement policies. The findings indicate that there is still substantial room for improving the hospitalization expense reimbursement rate of the migrant population, and it is necessary to further reduce barriers to their hospitalization expense reimbursement. Secondly, the migrant population tends to concentrate their hospitalization expense reimbursement in their household registration locations, with only 44.31% reimbursing in their inflow areas. In terms of reimbursement methods, 88.36% of the migrant populations opt for basic medical insurance for urban and rural residents to cover hospitalization expenses, while merely 11.64% choose basic medical insurance for urban workers. This is attributed to the higher proportion of the migrant population participating in basic medical insurance for urban and rural residents, whereas the proportion participating in basic medical insurance for urban workers stands at only 17.48%, signifying inequity in the migrant population's access to medical insurance. This shows that there is inequity in the medical insurance utilization by the migrant population, because the basic medical insurance for urban workers provides higher protection than the medical insurance for urban and rural residents. Eventually, the mean value of the level of hospitalization expense reimbursement for the migrant population is 3,058.7 (USD), and the reimbursement rate of medical insurance is only 39.67%, while the out-of-pocket payment rate of the migrant population is 60.33%. This shows that although China has implemented several health insurance reform measures to improve the convenience of hospitalization expense reimbursement for the migrant population and reduce their burden of medical care, such as direct settlement of medical expenses in different places, simplifying the reimbursement process, and narrowing the reimbursement gap between the place of enrollment and inflow, the hospitalization expense reimbursement for the migrant population still needs to be further strengthened.

The analysis results of the factors influencing whether or not the migrant population chooses to reimburse hospitalization expenses show that education, health, age, income, and location of insurance coverage are the five most critical key factors. Consistent with related studies (57), this study also finds a significant linear relationship between education and the probability of hospitalization expense reimbursement: migrant individuals with higher education levels are more likely to reimburse their hospitalization expenses. This may be because higher education not only helps expand access to expense reimbursement policies but also facilitates a better understanding of these policies, thereby reducing information asymmetry in the reimbursement process. However, as an important Predisposing factor in Andersen's health service utilization model, education significantly affects the migrant population's choices regarding hospitalization expense reimbursement, yet it is not easily alterable in the short term and functions more as a background variable. China's hospitalization expense reimbursement policies are relatively complex, with variations not only between different regions and healthcare insurance systems but also within the same insurance system regarding reimbursement policies for different tiers of medical institutions and coverage scopes. This further increases the difficulty of understanding and accepting these policies. Therefore, in order to further improve the hospitalization expense reimbursement rate of the migrant population in view of their lower education level, more emphasis can be placed on the accessibility of enabling resources, such as simplifying reimbursement policies and optimizing the information dissemination channels. This way, even among the migrant population with lower education levels, their ability to utilize healthcare services can be improved through system optimization. Migrant individuals in good health have a lower probability of reimbursing hospitalization expenses, probably because this group incurs relatively low hospitalization expenses, either failing to meet the reimbursement threshold or being able to afford the expenses and choosing to pay out of pocket. The impact of age on the choice of hospitalization expense reimbursement shows that the migrant population becomes more willing to reimburse expenses as they age, which aligns with existing research results (58). Compared with young people, middle-aged, and older adult individuals are in the middle and later stages of the life, a period when previously latent health issues may emerge, and their bodily functions decline with age, making them more susceptible to diseases (59). They also face higher hospitalization expenses, and the increased expense burden leads to a stronger willingness to seek reimbursement. The impact of income on hospitalization expense reimbursement is reflected in the fact that migrant populations with a middle income or higher are more likely to be reimbursed for hospitalization expenses. This study finds that although low income has a positive effect on hospitalization expense reimbursement, the effect is not significant, indicating that greater attention should be paid to improving expense reimbursement for this group. For example, measures such as lowering the hospitalization expense reimbursement threshold, increasing reimbursement benefits, and implementing the “treatment first, payment later” policy can reduce their expense burden. The location of insurance coverage is also an important factor influencing the migrant population's choice of hospitalization expense reimbursement, likely due to differences in reimbursement benefits between insurance purchased in the household registration location and the inflow area, as well as the complexity of cross-regional hospitalization expense reimbursement procedures, which restrict their reimbursement choices (60, 61). Although China has integrated its fragmented healthcare insurance system, there are still significant differences in reimbursement procedures and levels across regions. Therefore, to improve the hospitalization expense reimbursement level of the migrant population in the future, it is necessary not only to actively encourage them to participate in medical insurance in their inflow areas but also to further enhance the integration of medical insurance, promoting the upgrading of basic medical insurance from municipal to provincial coordination, thereby reducing regional differences in benefits for the migrant population.

The key factors affecting the hospitalization expense reimbursement level of the migrant population include health status, insurance type, total medical expenditure, illness, and mobility scope. Health status has a significant negative impact on reimbursement levels: different health statuses mean that migrant individuals face varying health risks, thus consuming different amounts of healthcare resources when hospitalized. For those in poor health, the increased consumption of health resources to maintain their health leads to higher hospitalization expenses, which in turn raises reimbursement amounts. Insurance type also positively affects the level of hospitalization expense reimbursement, due to significant differences in reimbursement levels between different types of health insurance. Participation in BMISURR can result in higher reimbursement compared to BMIUE. It is worth noting that to address this inequity in hospitalization expense reimbursement, the Chinese government's 2022 policy to further improve direct settlement of basic medical insurance for cross-regional medical treatment explicitly proposes to narrow policy differences between different systems in areas such as funding levels, reimbursement standards, and coverage catalogs. All of these reform measures have helped to narrow the inequities between different insurance systems and improve the expense coverage treatment of the migrant population. Total medical expenditure will have a significant positive impact on the level of expense reimbursement, which may be due to the fact that the Chinese health insurance system provides stronger protection for patients with large medical expenditures. For a long time, China's basic health insurance has shown a remarkable characteristic of emphasizing hospitalization protection, i.e., a higher level of protection for participants' hospitalization services, and the reimbursement rate for hospitalization is generally higher than that for small outpatient expenditures. Meanwhile, patients with high medical expenditures are more inclined to initiate reimbursement, and the actual incurred medical expenses are more likely to reach the threshold, thus triggering the health insurance payment mechanism. Therefore, as total medical expenditures increase, the level of expense reimbursement received by patients increases accordingly. However, this study also found that the current hospitalization expense reimbursement rate for the migrant population was still relatively low, which means that the proportion of hospitalization expenses paid by themselves is relatively high. Therefore, in the future, the hospitalization expense protection for the migrant population should be further strengthened to effectively reduce their medical expense burden. Illness has a significant negative impact on the level of expense reimbursement, This study found that illness has a significant negative impact on the level of hospitalization expense reimbursement for the migrant population, which may be closely related to the obstacles faced by the migrant population group in settling medical bills in different places, and the lower proportion of inter-regional reimbursement. People with chronic diseases or more serious conditions often need to be hospitalized in other places, but due to cumbersome referral procedures and information asymmetry, some patients fail to realize direct settlement, and need to make advance payments before reimbursement, resulting in a decline in the actual level of reimbursement. In addition, the structure of hospitalization expenses for the sick population is complex, with many out-of-pocket expenses, and a number of small hospitalizations have failed to fully meet the high reimbursement amount, further reducing the actual level of reimbursement of hospitalization expenses. Therefore, to further strengthen the hospitalization protection for the floating population in the future, on the one hand, the direct settlement system for medical treatment in other places can be improved, the filing and referral process can be simplified, and the accessibility and convenience of reimbursement for hospitalization in other places can be enhanced; on the other hand, it is also necessary to gradually narrow the differences in reimbursement ratios among different regions, so as to ensure that the floating population enjoys hospitalization protection treatments in the other places on an equal footing with those of the insured. The scope of mobility has a significant positive impact on the level of hospitalization reimbursement for the migrant population, and this study suggests that this may be due to the fact that the migrant population with a larger scope of mobility tends to be a group with a high degree of long-term mobility and stability of migration, and they tend to have a stronger willingness and ability to participate in the insurance scheme. Some individuals who have been moving across provinces for a long period of time may have completed the procedures of enrollment and transfer of employee or resident health insurance in the place of inflow, and thus can make direct settlement and high reimbursement when they are hospitalized in a different place. Secondly, people with greater mobility tend to have richer social networks and better access to information, and are better able to understand the policies on filing, hospitalization settlement and reimbursement for medical treatment in a different place; they may be more familiar with the reimbursement process, and their awareness of reimbursement and access to information is stronger, which will help to effectively lower the actual reimbursement obstacles brought about by medical treatment in a different place, and thus increase the actual level of reimbursement for hospitalization expenses.

In terms of reimbursement equity, this study's results show that the probability of hospitalization expense reimbursement and the level of reimbursement for the migrant population tends to favor the high-income groups, which is consistent with the results of other studies on the equity of hospitalization service utilization among China's migrant population (62). Notably, among the factors contributing to inequality in the probability of hospitalization expense reimbursement, education and income have the highest contributions, while the contribution of insurance participation location is the lowest. This indicates that to improve the migrant population's access to hospitalization expense reimbursement, more attention should be paid to factors other than health insurance. Among the factors contributing to inequality in hospitalization expense reimbursement levels, health status and mobility scope have the highest contributions, while the contribution of insurance type is only 9.28%. Currently, China has made important reforms to the health system and medical insurance system to improve the utilization of medical services and expense reimbursement for the migrant population, including the implementation of the policy of direct settlement of medical expenses for medical treatment in other places and the policy of provincial-level coordination of medical insurance, etc., which have improved the accessibility of expense reimbursement for the migrant population and safeguarded the health of the migrant population. The findings of this study on the contribution degree of different key factors to the migrant population's hospitalization expense reimbursement opportunities and levels will help provide a more detailed and scientific basis for further improving the equity of their hospitalization expense reimbursement in the future.

## Conclusion

Using data from the 2018 CMDS, this paper first analyzes the current situation of hospitalization expense reimbursement for the migrant population, then applies the Random Forest algorithm to evaluate the importance of factors influencing hospitalization expense reimbursement, quantitatively analyzes key variables using logistic regression, and finally uses the CI to identify the contribution degree of key variables. The main conclusions drawn are as follows: firstly, the hospitalization expense reimbursement rate of the migrant population needs to be further improved. Secondly, the migrant population mainly chooses to reimburse hospitalization expenses in their household registration locations, with a relatively low proportion reimbursing in inflow areas; meanwhile, they mainly rely on urban and rural residents' medical insurance for reimbursement, with insufficient use of urban workers' medical insurance. Thirdly, the migrant population's hospitalization expense reimbursement level and rate are relatively low. Fourthly, the key factors affecting whether the migrant population reimburses hospitalization expenses are education, health status, age, income, and local insurance participation; the key factors affecting their hospitalization expense reimbursement level are health status, insurance type, total medical expenditure, illness, and mobility scope. Fifthly, both the opportunities and levels of hospitalization expense reimbursement for the migrant population tend to favor high-income groups, with education, income, and health status being the main factors contributing to reimbursement inequality. To further strengthen the protection of hospitalization expenses for the migrant population in the future, the level of medical insurance should be further improved to narrow the treatment differences between different regions; the migrant population should be encouraged to participate in medical insurance in their inflow areas as well as to actively participate in the BMIUE, to increase the level of reimbursement for the expenses of the migrant population; and the reimbursement policy should be simplified and the reimbursement information dissemination channel optimized, to enhance the comprehensibility and acceptance of the policy, and narrow the gap in the accessibility of the policies to the floating population.

## Limitations

It is worth noting that there are still some limitations in this study, firstly, the data we use comes from secondary data of open dataset, due to the limitation of the data, this paper does not analyze the hospitalization expense reimbursement of the migrant population in different levels of healthcare institutions for the time being; at the same time, the time of the research of the data used in this study is 2018, and there is a certain lag in the data. In the future, with the opening of the latest data, the latest data can be further used to analyze the new characteristics of the reimbursement of hospitalization expenses of the migrant population. In addition, the data used in this study is a large-scale cross-sectional survey, based on which we explored the relationship between hospitalization expense reimbursement and key factors among the Chinese migrant population. However, we were unable to draw causal inferences from the available cross-sectional data. In the future, with the abundance of data, more recent and relevant datasets (e.g., Chinese General Social Survey, China Labor-force Dynamic Survey) or data from longitudinal studies can be used to further explore the relationship between different influencing factors and hospitalization expenses of the migrant population. Second, the cross-sectional design of this paper fails to demonstrate the long-term trend of hospitalization expense reimbursement for the migrant population. In the future, if longitudinal data over a long period are available, changes in the key factors affecting hospitalization expense reimbursement for the migrant population over time, as well as the dynamics of the equity of hospitalization expense reimbursement services, can be explored. Third, a large number of samples were deleted during the data cleaning process due to missing key variables such as hospitalization behaviors and hospitalization expenses, which resulted in a relatively small amount of cross-sectional data for this study, and this bias may have also affected the extrapolation of the findings, limiting the statistical validity of the findings, and resulting in the findings not being representative of the larger population. In addition, limited data may also affect the ability to detect significant effects or differences, leading to uncertain interpretations. As data become progressively more plentiful, future studies could use longitudinal data to expand the dataset, employ complementary statistical methods, or consider study designs that are better suited to data-limited situations to enhance the findings and improve their representativeness and scientific validity. Fourth, due to the data limitations of the 2018 CMDS, we were only able to utilize the indicator of whether a patient was ill as a proxy for the severity of their illness, and failed to control for indicators such as the type of illness and the severity of the illness; at the same time, the 2018 CMDS database also lacked information on the outpatient expenses of the respondents, which allowed this study to incorporate total medical expenditures in the covariate only by utilizing the total inpatient hospitalization expenses of the respondents to conduct the measurement. In the future, with the abundance of data, firstly, indicators such as disease type and disease severity can be included in the analysis, and secondly, outpatient expenses can also be further included in the scope of total medical expenditures to provide a more complete explanation of the influencing factors of the hospitalization expenses of the migrant population. Fifth, although CMDS has good representativeness, it also has certain limitations, such as respondents' subjectivity in answering the questionnaire, and the hospitalization expenses used in this study for the dependent variable were obtained by asking the respondents, and there may be bias in the hospitalization expenses obtained in this way, and in the future, with the enrichment of data, more objective methods can be adopted. Richness of the data, a more objective way of obtaining hospitalization expenses can be adopted to avoid the bias of respondents' subjective answers. In addition, this paper uses respondents' self-assessed health indicators to reflect the health status of individuals, but they are also subjective, and it is necessary to further enrich and improve the measurement indicators in the future, for example, the diagnosis and evaluation results of professional doctors can be taken as an objective measure of health level. Sixth, the study analyzed the influencing factors of health education for the migrant population using the random forest model and logistic regression method, and the combination of the two methods can help to overcome the shortcomings of a single method, but this study has not yet used other machine learning methods, such as decision trees and XGBoost, etc., and in the future, these methods can be considered for in-depth research on the reimbursement of hospitalization expenses for the migrant population, to promote the health status of migrant populations. In-depth research can be conducted to provide more evidence to support the promotion of the health of the migrant population. Seventh, this study found that factors in Andersen's model (e.g., education) have a role in explaining hospitalization expense reimbursement for the migrant population, but their immutability determines limited space for policy intervention. Future research should focus more on how to strengthen hospitalization expense coverage for the migrant population by improving enabling resources and meeting need factors.

## Data Availability

Publicly available datasets were analyzed in this study. This data can be found here: data supporting the results of this study came from the National Mobile Population Dynamics Monitoring Survey (CMDS). The datasets generated and/or analyzed during the current study are available in the official website (https://www.ncmi.cn/project/project-showProjectList.html?type=data&amp;id=d3).
